# Interplay between Humoral and CLA^+^ T Cell Response against *Candida albicans* in Psoriasis

**DOI:** 10.3390/ijms22041519

**Published:** 2021-02-03

**Authors:** Carmen de Jesús-Gil, Lídia Sans-de San Nicolàs, Ester Ruiz-Romeu, Marta Ferran, Laura Soria-Martínez, Irene García-Jiménez, Anca Chiriac, Josep Manel Casanova-Seuma, Josep Manel Fernández-Armenteros, Sherry Owens, Antonio Celada, Michael D. Howell, Ramòn María Pujol, Luis Francisco Santamaria-Babí

**Affiliations:** 1 Translational Immunology, Department of Cellular Biology, Physiology and Immunology, Faculty of Biology, Universitat de Barcelona, 08028 Barcelona, Spain; cdejesus@ub.edu (C.d.J.-G.); li.sns25@gmail.com (L.S.-d.S.N.); eruizromeu@gmail.com (E.R.-R.); laurasoriamartinez@gmail.com (L.S.-M.); irenegarciajimenez@ub.edu (I.G.-J.); 2 Department of Dermatology, Hospital del Mar, 08003 Barcelona, Spain; mferran@parcdesalutmar.cat (M.F.); rpujol@parcdesalutmar.cat (R.M.P.); 3 Department of Dermatophysiology, Apollonia University, 700613 Iasi, Romania; ancachiriac@yahoo.com; 4 Department of Dermatology, Hospital Universitari Arnau de Vilanova, 25198 Lleida, Spain; josepmanel.casanova@udl.cat (J.M.C.-S.); josepmanelfa27@gmail.com (J.M.F.-A.); 5 Translational Sciences, Incyte Corporation, Wilmington, DE 19803, USA; SOwens@incyte.com (S.O.); mhowell@dermtech.com (M.D.H.); 6 Macrophage Biology, Department of Cellular Biology, Physiology and Immunology, Faculty of Biology, Universitat de Barcelona, 08028 Barcelona, Spain; acelada@ub.edu

**Keywords:** psoriasis, *Candida albicans*, IgA, CLA, IL-17

## Abstract

*Candida albicans* (CA) infections have been associated with psoriasis onset or disease flares. However, the integrated immune response against this fungus is still poorly characterized in psoriasis. We studied specific immunoglobulins in plasma and the CA response in cocultures of circulating memory CD45RA^−^ cutaneous lymphocyte antigen (CLA)^+/−^ T cell with autologous epidermal cells from plaque and guttate psoriasis patients (cohort 1, *n* = 52), and also healthy individuals (*n* = 17). A complete proteomic profile was also evaluated in plaque psoriasis patients (cohort 2, *n* = 114) regarding their anti-CA IgA levels. Increased anti-CA IgA and IgG levels are present in the plasma from plaque but not guttate psoriasis compared to healthy controls. CA cellular response is confined to CLA^+^ T cells and is primarily Th17. The levels of anti-CA IgA are directly associated with CLA^+^ Th17 response in plaque psoriasis. Proteomic analysis revealed distinct profiles in psoriasis patients with high anti-CA IgA. C-C motif chemokine ligand 18, chitinase-3-like protein 1 and azurocidin were significantly elevated in the plasma from plaque psoriasis patients with high anti-CA levels and severe disease. Our results indicate a mechanism by which *Candida albicans* exposure can trigger a clinically relevant IL-17 response in psoriasis. Assessing anti-CA IgA levels may be useful in order to evaluate chronic psoriasis patients.

## 1. Introduction

Psoriasis is a T cell-mediated skin disease resulting from epithelial and immunological cells’ interactions [1]. Psoriasis onset is influenced by genetic and environmental factors, particularly infections. Psoriasis flares have been attributed to *Candida albicans* infections [2]. Several studies have confirmed increased *Candida albicans* colonization of the oral [3,4,5,6,7] and gut [3,7] mucosa in psoriasis patients compared to healthy individuals. Whereas some authors revealed an association between *C. albicans* existence at mucosal sites and psoriasis severity [3,5,7], others did not find a correlation [4,8,9]. Likewise, the presence of *Candida albicans* at cutaneous levels is still controversial. Most skin mycobiome studies have not revealed differences between psoriasis and healthy individuals’ Candida spp. levels [4,10,11,12,13,14,15,16]. However, Sarvtin T. and colleges found elevated *C. albicans* in lesional skin from the trunk compared to either adjacent normal skin or healthy controls’ skin [6]. Furthermore, Salem I. et al. recently reported the presence of higher *C. albicans* in non-lesional skin swabs [17].

Currently, the evidence of microbes’ presence in psoriatic patients is focused on complex DNA-based technologies [18]. Specific immunoglobulins assess environmental microorganism exposure. The isotypes observed in immunoglobulins can elucidate the mechanisms of antigen encounters [19]. Initial studies of anti-Candida antibody levels did not observe differences between patients with psoriasis and healthy individuals [10,20]. Recently, however, Liang YS et al. showed increased anti-whole cell antigen IgG and decreased anti-soluble antigen IgA and IgM in serum from psoriasis patients versus healthy individuals [21]. However, Sarvtin MT et al. reported decreased anti-*Candida* IgM, IgA and IgG levels in psoriasis patients compared to controls [6]. As such, the humoral response against *Candida albicans* in psoriasis patients remains controversial.

The IL-23/IL-17/IL-22 axis protects us against *Candida albicans* [22], but it also plays a key role in psoriasis development. The study of *Candida albicans* in psoriasis patients is very limited and mainly studied in total peripheral blood mononuclear cells [23,24,25]. Schlapbach et al. reported IL-9 induction by *C. albicans* specifically on skin-tropic T helper cells from healthy donors, along with increased IL-9^+^ cells in psoriasis lesions [26]. Likewise, previous data from our group described the increased induction of IL-9, IL-17A and IFN-γ by CLA^+^CD4^+^ T cells after *C. albicans* activation in four psoriasis patients compared to healthy individuals [27]. Investigating CD45RA^−^CLA^+^ memory T cell-induced IL-17 responses to a microorganism with microbe-specific immunoglobulins levels with clinical features can help to understand how microorganisms might modulate psoriasis pathogenesis from a more holistic view. We have recently demonstrated the relevance of this approach for *Streptococcus pyogenes*, showing that bacteria specific-IgA and CLA^+^ T cell IL-17 response are associated in psoriasis patients [28].

In this study, we investigated humoral and cellular immune responses against *Candida albicans* in psoriasis patients, comparing plaque and guttate forms. The association between antibodies and cytokine responses was also assessed. Finally, we examined the proteomic profile in plasma from plaque psoriasis patients. Altogether, our data illustrate the heterogeneity of *Candida albicans* exposure in psoriasis, which might reflect the pathological mechanisms.

## 2. Results

### 2.1. Description of Patients and Controls Samples Used in Different Experiments

A total of 166 psoriasis patients and 17 healthy individuals were enrolled. Psoriasis patients were classified in cohort 1 (*n* = 52), consisting of both plaque and guttate forms of the disease, and cohort 2 (*n* = 114), which included only plaque psoriasis patients. Complete humoral and cellular responses against *Candida albicans* were assessed in cohort 1, as well as ELISAs to validate differentially expressed proteins. Cohort 2 was only studied for *C. albicans*-specific IgA levels and the complete proteomic profiling in plasma. Detailed information of the source and experiments performed with each cohort can be found in Supplementary Table S1. The inclusion and exclusion criteria for each cohort are detailed in Section 4.

### 2.2. Plaque Psoriasis Patients Have Increased Levels Candida Albicans-Specific IgA and IgG

**Figure 1:**
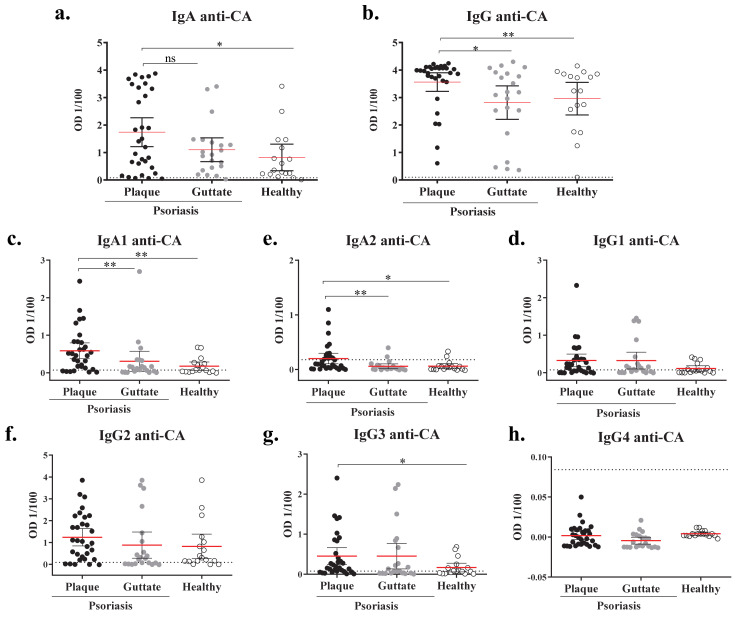
Plaque, but not guttate, psoriasis patients present increased IgA-1, IgA-2 and IgG-3 against *Candida albicans* compared to healthy controls. Specific immunoglobulins recognizing *C. albicans* cellular antigens (CA) were detected through ELISA in plasma collected from the blood of plaque psoriasis (*n* = 31, ●), guttate psoriasis (*n* = 21, ●) and healthy controls (*n* = 17, ○). Net OD of 1/100 diluted plasma is reported. Plasma levels of IgA (**a**), IgA-1 (**b**), IgA-2 (**c**), IgG (**d**), IgG1 (**e**), IgG2 (**f**), IgG3 (**g**) and IgG4 (**h**) against CA are shown. Statistics lines are represented as mean with 95% confidence interval. Dotted lines indicate the mean background signal. A Mann–Whitney test was used to compare between groups, and *p* values are indicated as: ns: *p* > 0.05; *: *p* < 0.05; **: *p* < 0.01.

**Figure 2:**
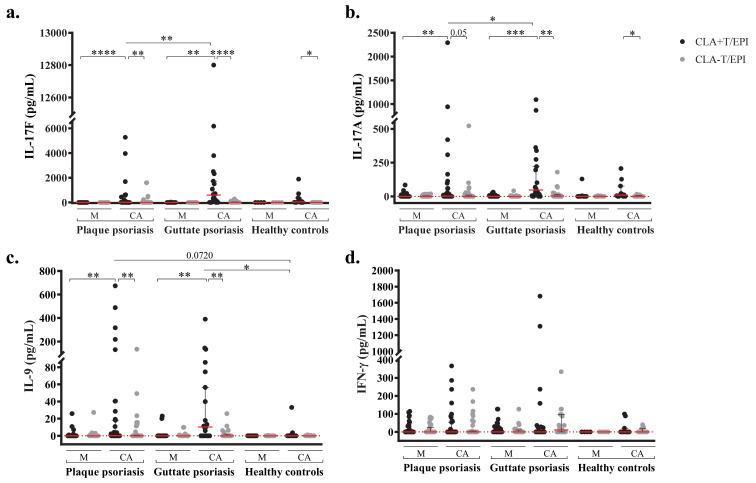
Th17 and Th9 responses to *C. albicans* are confined to CLA^+^ T cells. IL-17F (**a**), IL-17A (**b**), IL-9 (**c**) and IFN-γ (**d**) levels were measured in culture supernatants from CLA^+^ or CLA^−^ T cells cocultured with autologous epidermal cells (EPI) after 5 days, in basal conditions (M) and stimulated with *C. albicans* (CA), in plaque psoriasis (*n* = 31), guttate psoriasis (*n* = 21) and healthy controls (*n* = 12). Column bars are represented as mean with 95% confidence interval. Wilcoxon test was used to compare two conditions within psoriasis or controls (double-pointed line), whilst Mann–Whitney test was used to compare psoriasis versus controls (single-pointed line). For both, *p* values are indicated as ns: *p* > 0.05; *: *p* < 0.05; **: *p* < 0.01; ***: *p* < 0.001; ****: *p* < 0.0001.

Plasma samples from psoriasis cohort 1 (*n* = 52) and healthy (*n* = 17) individuals were analyzed. The clinical features of plaque and guttate psoriasis patients from cohort 1 are summarized in Table 1. The specific IgA and IgG for *Candida albicans* in plasma samples were tested by ELISA (referred as anti-CA IgA or IgG). The levels of anti-CA IgA and IgG were significantly increased in the plasma from plaque psoriasis patients compared to guttate psoriasis and healthy controls, which presented similar anti-CA IgA and IgG levels (Figure 1a,d). IgA and IgG subtypes were also assessed. Both anti-CA IgA1 and IgA2 were significantly increased in plaque psoriasis patients in comparison to guttate psoriasis and healthy individuals (Figure 2b,c), whereas no differences were observed between guttate psoriasis and controls. Interestingly, anti-CA IgA and IgA2 levels positively correlated with disease duration only in plaque psoriasis (IgA Spearman r: 0.4435, *p* value = 0.03; IgA2 r = 0.4082, *p* value = 0.0477, data not shown). Anti-CA IgG1, IgG2 and IgG3 were detected in both psoriatic groups and healthy individuals (Figure 1e–g), whereas anti-CA IgG4 was not detectable (Figure 1h). Nonetheless, only anti-CA IgG3 levels were significantly increased in plaque psoriasis compared to controls (Figure 1g). Additionally, we used a commercial diagnostic kit for the quantitative determination of IgA antibodies against *C. albicans* in human plasma, revealing current infection. Both psoriasis and healthy individuals presented negative antibody titer (< 8 U/mL), indicating no fresh *C. albicans* infection (Figure S1). Interestingly, plaque psoriasis patients showed significantly higher anti-CA titer compared to healthy controls, supporting our results.

### 2.3. Candida Albicans-Induced Th17 and Th9 Responses Are Confined to CLA+ T-cells and Dominated by IL-17F

*C. albicans* cellular response was assessed in psoriasis cohort 1 (*n* = 52) and healthy controls (*n* = 17). Cocultures of CD45RA^−^ memory CLA^+^ or CLA^−^ T cells with autologous epidermal cells were left untreated or activated with *C. albicans* extract. After 5 days of culture, cytokines were quantified in stimulated supernatants. Plaque and guttate psoriasis patients showed significant CLA^+^ T cells-dependent CA-mediated induction of IL-17F, IL17A, and IL-9 compared to unstimulated CLA^+^T/EPI and CA-stimulated CLA^−^T/EPI cocultures (Figure 2a–c). Notably, CA-induced IL-17F and IL-17A levels in CLA^+^T cells were significantly higher in cocultures from guttate psoriasis compared to those of plaque psoriasis. CA induction of IFN-γ was also higher in guttate psoriasis, whereas IL-9 induction was slightly increased in plaque psoriasis, but not significantly differently. Cytokine responses were also detected in healthy controls’ CLA^+^T/EPI cocultures after CA activation. Only IL-17F and IL-17A responses were significantly associated with CLA^+^ T cells when compared to CA-stimulated CLA^−^T/EPI coculture in healthy subjects; however, no substantial differences were found when compared to the CLA^+^T/EPI untreated condition. Interestingly, levels of CA-induced IL-9 by CLA^+^T/EPI cocultures were significantly higher in psoriasis than in healthy individuals (Figure 2d). Additionally, single cultures of epidermal cells as well as CLA^+^ and CLA^−^ T cells were left untreated and stimulated with CA for 5 days. IL-17A and IFN-γ induction was measured in culture supernatants. The IL-17A response was almost null even in CA-stimulated conditions (Figure S2a). Similarly, although low IFN-γ levels were detected in CA-stimulated conditions, still this response was similar to the one found in untreated cultures (Figure S2b). Therefore, only when CLA^+^ T cells are cultured in the presence of autologous epidermal cells is increased cytokine response to *C. albicans* stimulation observed.

### 2.4. Candida Albicans’ Specific IgA Plasma Levels and IL-17 T cell Responses Are Directly Associated in Plaque Psoriasis Patients

Correlations between anti-CA IgA or IgG and the different measured cytokines were assessed in psoriasis cohort 1 (*n* = 52) and healthy controls (*n* = 17). Only for plaque psoriasis patients were levels of anti-CA IgA positively associated with IL-17F and IL-17A responses (Table 2), in CLA^+^ T cells/EPI but also in CLA^−^ T cells/EPI cocultures. This association was not observed for guttate psoriasis (data not shown) or healthy controls, or for specific IgG (Table 2). Additionally, disease duration was significantly correlated with CA-induced CLA^+^ T cell dependent IL-17F and IL-17A, and CLA^−^ T cell dependent IL-17F production in plaque psoriasis (CLA^+^ T-IL-17F: r = 0.5569, *p* = 0.0047; CLA^−^ T-IL-17F: r = 0.4112, *p* = 0.0459 and CLA^+^ T-IL-17A: r = 0.5873, *p* = 0.0026).

### 2.5. Proteomic Profile of Plasma from Psoriasis Patients according to Anti-CA IgA Levels

**Figure 3:**
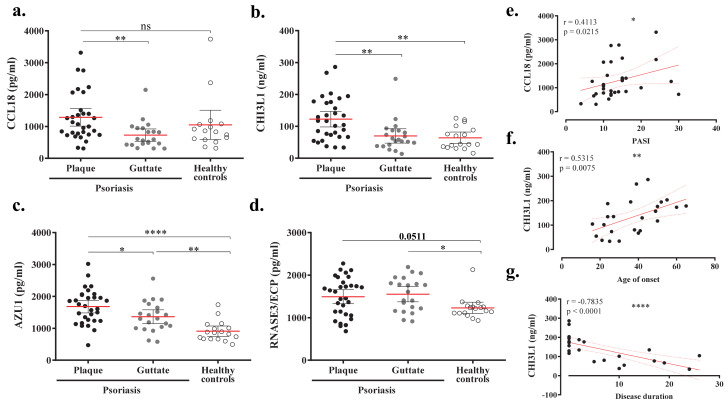
AZU1, CCL18, CHI3L1 and AZU1 are significantly increased in plasma from plaque psoriasis compared to guttate or/and healthy controls. CCL18, CHI3L1, AZU1 and RNASE3/ECP levels were assessed by ELISA in plasmas from plaque psoriasis (*n* = 31, ●), guttate psoriasis (*n* = 21, ●) and healthy individuals (*n* = 16, ○). (**a**–**d**) Comparison of levels detected between plaque and guttate psoriasis versus controls. In plaque psoriasis patients, relevant correlations between (**e**) CCL18 and disease severity (PASI), as well as CHI3L1 and age of onset (**f**) or disease duration (**g**), were found. A Wilcoxon test was used to compare two conditions within psoriasis or controls. Spearman test was used for correlations. Spearman coefficient (r) and *p* values (*p*) are indicated as ns: *p* > 0.05; *: *p* < 0.05; **: *p* < 0.01; ****: *p* < 0.0001.

Specific IgA was assessed in plasma samples from psoriasis cohort 2 (*n* = 114). Based on the upper limit of the 95% confidence interval and the maximum optical density (OD) signal of anti-CA IgA from healthy individuals, patients were stratified in the following groups: low (OD < 1.5), intermediate (OD = 1.5–3) and high (OD > 3). Broad proteomic expressions of 1012 proteins were compared between groups using a generalized linear model. Table S2 shows the differentially expressed proteins between low and high groups ordered by fold change. Positive fold change values represent higher protein levels in the high IgA-Candida group. A total of 27 proteins differed significantly between the high and low groups using a raw *p* value. Of these, two proteins yielded >30% of values below the limit of detection and should be interpreted with caution. Although no significant differences remained after false discovery rate (FDR) *p* value correction, some proteins of potential interest were identified and selected for further validation in samples from cohort 1 (*n* = 52) and healthy individuals (*n* = 17). Four proteins were found with increased presence in plasmas with high anti-CA IgA (eosinophil cationic protein (RNASE3/ECP), chitinase-3-like protein 1 (CHI3L1), azurocidin (AZU1) and C-C motif chemokine 18 (CCL18)), and two that were found to be decreased (Follistatin (FST) and Fas-ligand (FSLG)). Plasma levels of CCL18, CHI3L1 and AZU1 were significantly increased in plaque compared to guttate psoriasis (Figure 3a–d). CHI3L1 levels were significantly higher in plaque but not guttate psoriasis when compared to controls (Figure 3b). Similar results were obtained for CCL18, although differences between plaque psoriasis and healthy individuals did not reach statistical significance (Figure 3a). Conversely, AZU1 and RNASE/ECP protein levels were significantly higher in both plaque and guttate psoriasis compared to healthy subjects (Figure 3c,d). Plaque psoriasis patients showed significant positive correlation between CCL18 levels and PASI, as well as between CHI3L1 levels and age of onset (Figure 3e,f), whereas a strong negative correlation was found for CHI3L1 and disease duration (Figure 3g). In psoriasis patients, we also found significant direct correlations between levels of CCL18, CHI3L1 or AZU1 and PASI, as well as age of onset (Figure S3). No differences were observed for FST and FSLG (data not shown).

## 3. Discussion

Microorganisms can affect psoriasis presentation and patient’s natural history [2,29]. While lymphoid B-cells are dispensable for psoriasis development, B-cell dysregulation and increased IgA has been shown in psoriasis [30]. Our results showed that non-treated plaque psoriasis patients present increased plasma levels of *Candida*-specific IgA that correlate with the IL-17 response to this fungus in vitro.

To evaluate *Candida albicans* exposure in psoriasis patients, fungus-specific immunoglobulins in plasma were assessed. Significantly elevated levels of IgA and IgG against *Candida albicans* were found in the plasma from plaque psoriasis patients, particularly those with higher disease severity, compared to healthy controls. Current studies of *Candida albicans*-specific immunoglobulins in psoriasis are controversial. One study reported increased anti-whole cell antigen IgG but decreased anti-soluble antigen IgA and IgM in psoriasis vulgaris versus healthy controls [21]. More recently, generally reduced anti-CA immunoglobulins plasma levels in psoriasis vulgaris were shown, with no association with disease severity or duration [6]. Importantly, 76% of their psoriasis cohort had mild to moderate forms of the disease, which may explain the low levels of antibodies detected compared to our results. To better characterize anti-*C. albicans* antibody response, different subtypes of IgA and IgG were assessed. Both anti-CA IgA1 and IgA2 plasma levels were increased in plaque psoriasis patients compared to guttate psoriasis and controls. Human monomeric IgA subclasses are predominantly secreted by IgA-producing cells in the bone marrow, but also in the spleen, synovial tissue and gingiva [31]. Specifically, IgA1-secreting cells are present in the upper orogastric tract, whereas IgA2-secreting cells predominate in the lower gastrointestinal tract [19]. B-cells activated in the gut-associated lymphoid tissue (GALT) may be home to the marginal zone of the spleen [32]. Therefore, the commensal or pathogenic-specific IgA produced by these GALT activated-plasma cells may be secreted into the bloodstream. Despite being a commensal fungus, the presence of higher levels of *Candida albicans* in both oral and gut samples from psoriasis patients is widely demonstrated [33]. Altogether, our results identify both the oral and gut mucosa as sites of encounter with *C. albicans* and IgA production. However, we cannot exclude the possible existence of additional sources of CA-specific IgA in psoriasis, since its prevalence in cutaneous tissues is not well-defined, and skin-homing IgA1- and IgA2-secreting cells have also been described [19].

Memory CLA^+^ T cells participate in cutaneous immune responses due to their skin-homing properties [34]. A preferential CLA^+^ T cell response to *Candida albicans,* dominated by IL-17F, was found in psoriasis but also in control subjects. Although preferred *C. albicans* induction of IL-9 on CLA^+^ T cells has already been shown in healthy donors [26], we observed significantly increased CA-induced IL-9 response in psoriasis patients compared to controls. We found differentiated T-cell responses to *C. albicans* stimuli between plaque and guttate psoriasis, indicating increased cytokine responses in the latter. This finding is of special interest since the IL-23/Th17 axis drives and maintains psoriasis pathogenesis [1] and *Candida albicans* may fuel the IL-17/Th17 loop, thus perpetuating psoriasis. Of note, a recent study observed that responses to commensal skin fungi, such as *Candida albicans,* enhance psoriasiform Th17 inflammation in the imiquimod-induced psoriatic mice model [35].

Interestingly, we reported different humoral and cellular responses to *Candida albicans* plaque and guttate psoriasis. Whereas *C. albicans*-specific antibody levels are increased in the former, the T-cell response to *C. albicans* extract is higher in the latter. This may be due to disparities in the course of each type of disease. While guttate psoriasis occurs in a more acute way and generally has better prognosis, plaque psoriasis is usually more severe, harder to control, and therefore, presents a more chronic duration. Plaque psoriasis patients are more likely to receive immunosuppressive treatment intermittently and for longer periods of time, which would cause microbiome dysbiosis, leading to increased *C. albicans* colonization and an increased presence of specific IgA in plasma. Nevertheless, because guttate psoriasis occurs more abruptly, skin and blood samples are usually taken closely to the appearance of the flare, wherein pro-inflammatory CLA^+^ T cells in circulation are actively recruited to the cutaneous tissue. Conversely, in plaque psoriasis patients, the recirculation of pro-inflammatory T-cells may diminish or rebalance over time, resulting in a continuous flow of minor cells towards cutaneous lesions. This distinct presence of active pro-inflammatory T-cells in circulation may explain the substantial response to CA extract in cocultures from guttate psoriasis patients.

The link between Th17 and IgA has been extensively studied in animal models [36,37] and humans. Although the molecular mechanisms underlying this effect are not fully understood, Shibui A et al. suggested an indirect role for IL-17 in antibody production, possibly by enhancing B-cell activators by other immune cells [38], whilst Ferreti E and colleagues demonstrated how B-cell migration within germinal centers is modulated by the IL-17A/IL-17RA axis [39]. A direct correlation between anti-CA IgA levels and IL-17 cytokines responses is found for both CLA^+^ and CLA^−^ T only in cocultures from plaque psoriasis patients. Recently, Wilson et al. reported the presence of antibody-secreting cells (ASC) in healthy skin, secreting mainly IgM but also IgA and IgG, which are important for maintaining tissue homeostasis [40]. The role of skin-associated B-cells in homeostasis has also been recently reviewed [41], highlighting their interplay with other skin-resident immune cells. We hypothesized that skin-associated B-cells may present *Candida albicans* peptides to antigen-specific CLA^+^ T inducing its activation and fungus-specific antibody secretion. Recently published data from our group demonstrated a similar correlation between *S. pyogenes*-specific IgA and CLA^+^ T-cell IL-17 response in psoriasis [28]. However, despite the direct correlation of the in vitro cytokine response to each antigen, no effect of the association between psoriasis patients’ exposure to *S. pyogenes* and *C. albicans* on IgA/G levels has been observed (data not shown). Nonetheless, IL-17 response by CLA^−^ T cells may contribute to *C. albicans*-specific IgA generation at extracutaneous sites, as mentioned above.

Patients with high anti-CA IgA levels may carry pathophysiological peculiarities, creating disease heterogeneity. Despite the fact that differentially expressed proteins were not statistically significant after FDR correction, which remains a limitation of our study, patients with high anti-CA IgA displayed generally increased levels of proteins involved in antimicrobial humoral response, cell chemotaxis and inflammatory immune response. Human RNASE3 is a cytotoxin with high anti-candida activity [42]. CHI3L1, which is regulated by IL-17F/A [43,44], has also shown anti-candida properties [45]. Azurocidin, secreted by neutrophils, acts against *Candida albicans* [46], and has been reported as a biomarker for periodontal disease [47]. CCL18 mediates CLA^+^ memory T cells homing towards skin [48]. Its expression is not only upregulated in lesions from atopic dermatitis and psoriasis [49,50], but also gingival biopsies from periodontitis [51]. Increased CCL18, CHI3L1 and AZU1 levels were detected in plasmas from plaque psoriasis, but we could not confirm their association with anti-CA IgA levels, which remains a limitation of the study. Our data support evidence linking the presence of *C. albicans,* psoriasis pathogenesis and periodontal disease [52,53,54]. Nonetheless, longitudinal follow-up studies are required to better establish a causal relationship.

We believe psoriasis patients’ increased cellular and humoral responses to *C. albicans* appear as a consequence of increased exposure to this fungus. Th17 response is fundamental for fighting against *Candida* spp. infection [55], as it is key for psoriasis immunopathogenesis. Therefore, we hypothesize that our observation could be related to treatment approaches for psoriasis. Effective therapies aim to reduce Th17 response in patients that may facilitate fungal colonization and the subsequent promotion of IL-17 responses, thus fueling psoriasis pathogenesis in a vicious circle. This hypothesis deserves careful analysis in future clinical studies. On the other hand, a predisposing background to increased response to *C. albicans* in psoriasis cannot be ruled out. Single nucleotide polymorphisms on IL-17 and IL-23 related genes, described as psoriasis genetic risk makers, could be associated to altered responses to *Candida* spp., a matter that remains unexplored to our knowledge.

In summary, the presence of IgA against *Candida albicans* in plasma from patients with plaque psoriasis without clinical signs of infection identifies subjects that have been exposed to this microbe, which preferentially activates skin-homing CLA^+^ T cells to secrete IL17F and IL17A, two cytokines that are clinically demonstrated to be relevant in psoriasis immunopathogenesis. We consider that *Candida albicans* exposure may affect psoriasis evolution, and eventually response to therapies. Assessing anti-CA IgA levels may be beneficial to better evaluate and stratify psoriasis patients.

## 4. Materials and Methods

### 4.1. Patients

All participants contributed voluntarily and provided written informed consent, and human material collection has been approved by the corresponding Ethical Committees. Psoriasis cohort 1 included non-treated patients with plaque or guttate lesions, without any age or sex restriction. Exclusion criteria included any systemic treatment during the 4 weeks prior to the study or any topical treatment during the last 2 weeks. Psoriasis cohort 2 comprised baseline plasma samples from the Clinical Trial NCT00778700, which included plaque psoriasis involving 2 to 20% body surface area, and those aged from 18 to 75 years. Exclusion criteria included lesions solely involving intertriginous areas, the scalp or the face, pustular psoriasis or erythroderma, systemic therapy, being currently on other topical agents or UVB within 2 weeks of the first dose of study medication, having started or discontinued therapy within 2 months of screening with agents that can exacerbate psoriasis, or currently receiving systemic triazole antifungals.

### 4.2. ELISA

ELISAs were performed as previously described [28]. The antibodies are listed in Supplementary Table S2. As the negative control of the experiment, CA-coated wells were incubated with PBS-1% skimmed milk instead of plasma dilution. The titer of reactive anti-CA antibody was taken as the OD_405nm_ after background signal (OD_570nm_) and negative control well signal subtraction, referred to as net OD 1/100. As a positive control of the technique, the wells were coated with human IgA/G isotype controls (Invitrogen, Carlsband, CA, USA), incubated with PBS as the primary antibody and a corresponding secondary antibody before substrate addition.

### 4.3. Circulating Memory T-cell and Epidermal Cell Isolation

Peripheral blood mononuclear cells were isolated by Ficoll gradient (GE Healthcare, Princeton, NJ, USA) and, after subsequent immunomagnetic separations (Miltenyi Biotech, Bergisch Gladbach, Germany), memory CD45RA^−^ CLA^+^ and CLA^−^ T cells were purified as previously described [56]. Punch skin biopsies (4–6 mm) were incubated overnight in dispase (Corning, Corning, NY, USA) at 4 °C, then the epidermal sheet was peeled off from the dermis. The epidermis was cut in pieces and incubated in trypsin (Biological Industries, Kibbutz Beit Haemek, Israel) for 15 min at 37 °C. Epidermal tissue was then mechanically disaggregated by gently pipetting and the cell suspension was transferred to fresh culture media (RPMI, 10% FBS, 1% penicillin-streptomycin (SIGMA-Aldrich, St. Louis, MO, USA)).

### 4.4. Co-Cultures

The ex vivo cocultures consisted of 5 × 10^4^ CLA^+/−^ T-cells plated together with 3 × 10^4^ autologous epidermal cells at the same time (CLA^+^T/Epi or CLA^−^T/Epi, respectively), seeded together simultaneously in 96-well flat-bottom plates (SIGMA-Aldrich, St. Louis, MO, USA), in the culture media described above. Cocultures were left untreated or activated with CA extract (Stallergenes Greer, Lenoir, NC, USA) reconstituted at 800 μg/mL in sterile water (SIGMA-Aldrich). CA extract was used at 20 μg/mL final well concentration. After 5 days of culture, supernatants were collected and kept frozen at −20 °C for later cytokine quantification.

### 4.5. Cytokine Quantification

Multiplex fluorescent bead-based immunoassays were used to measure IL-17A and IFN-γ (Diaclone SAS, Besançon, France) and IL-17F (BD Bioscience, Franklin Lakes, NJ, USA) concentration in collected culture supernatants. IL-9 concentration was quantified by using pre-coated ELISA kits (Biolegend, San Diego, CA, USA).

### 4.6. Proteomic Study

Anti-CA IgA plasma levels were measured in 114 plasma samples collected at baseline from psoriasis cohort 2. Patients were stratified into high (>3 OD, *n* = 61), intermediate (1.5–2.9 OD, *n* = 26), and low (<1.5 OD, *n* = 27) IgA-Candida. Broad proteomic analysis of plasma samples was conducted by OLINK Proteomics (*n* = 1012 proteins) (Watertown, MA, USA) and compared between groups using a generalized linear model. For technical information go to www.olink.com/downloads. Least-squared mean differences were used to compare high vs. negative groups. Statistical analyses were conducted in Array Studio version 10.1. Three of the differentially expressed proteins were selected for validation in a different cohort of patients (cohort 1, *n* = 52) and healthy individuals (*n* = 16) via ELISA (Fine test, Wuhan Fine Biotech Co., Ltd., Wuhan, China): RNASE3, AZU1, CHI3L1, CCL18, FST and FSLG.

### 4.7. Statistical Analysis

GraphPad Prism software (Version 8, GraphPad Software Corporation, San Diego, CA, USA) was used for statistical analysis and graphical representation. Data are generally represented as the mean and 95% confidence interval (CI). Differences between two conditions within the same group were analyzed by Wilcoxon test and represented by double pointed lines. Differences between two groups were analyzed by the Mann–Whitney test and represented by single pointed lines. Differences were considered significant at a *p* value of less than 0.05 and represented by the following symbols: (ns): *p* > 0.05; (*): *p* < 0.05; (**): *p* < 0.01 and (***): *p* < 0.001. The correlations were examined using Spearman’s rank correlation, and the Spearman’s coefficient and *p* value are indicated for each test.

## Figures and Tables

**Table 1:** Clinical features of psoriasis patients in cohort 1 according to the subtype of disease.

		Plaque	Guttate	*p* Value
**Number of patients**		31	21	NA
**Age (mean ± SD)**		46.65 (11.97)	32.65 (10.92)	***
**PASI (mean ± SD)**		13.74 (5.99)	7.23 (2.91)	***
HLA-Cw*6 **% (*n*)**	**Positive**	32.26 (10)	85.71 (18)	NA
	**Negative**	61.29 (19)	14.29 (3)	NA
	**UK**	6.45 (2)	-	NA

Mann–Whitney test was used to compare quantitative variables, *p* values are indicated as ns: *p* > 0.05; ***: *p* < 0.001. NA = not assigned. Bold: the clinical features.

**Table 2:** Correlation of anti-CA IgA or IgG and cytokine responses to *Candida albicans* in CLA^+^ or CLA^−^ T cells cocultured with autologous epidermal cells. Spearman ρ and *p* values are indicated (ns: *p* > 0.05; *: *p* < 0.05; **: *p* < 0.01).

			Anti-CA IgA			Anti-CA IgG		
	Coculture Condition	Cytokine	Spearman ρ	*p* Value		Spearman ρ	*p* Value	
**Plaque psoriasis (*n* = 31)**	CLA^+^T/EPI	IL-17F	**0.3735**	**0.0385**	*	−0.0842	0.6525	ns
		IL-17A	**0.4798**	**0.0063**	**	0.0349	0.8521	ns
		IL-9	0.2363	0.2006	ns	0.1250	0.5028	ns
		IFN-γ	0.1872	0.3219	ns	0.0116	0.9515	ns
	CLA^−^T/EPI	IL-17F	**0.5028**	**0.0039**	**	−0.0267	0.8864	ns
		IL-17A	**0.4714**	**0.0074**	**	0.0625	0.7383	ns
		IL-9	0.2558	0.1724	ns	0.1183	0.5264	ns
		IFN-γ	−0.0073	0.9690	ns	−0.0589	0.7573	ns
**Healthy controls (*n* = 12)**	CLA^+^T/EPI	IL-17F	−0.4496	0.1681	ns	0.0458	0.9015	ns
		IL-17A	−0.4307	0.1622	ns	−0.0392	0.9061	ns
		IL-9	−0.5691	0.0591	ns	0.0734	0.8288	ns
		IFN-γ	−0.2203	0.5015	ns	−0.3885	0.2227	ns
	CLA^−^T/EPI	IL-17F	0.2197	0.5192	ns	0.2197	0.5192	ns
		IL-17A	−0.1706	0.5948	ns	0.2538	0.4246	ns
		IL-9	−0.2527	0.4394	ns	0.0161	0.9697	ns
		IFN-γ	−0.0275	0.9394	ns	0.4773	0.1258	ns

## Data Availability

The data presented in this study are available on request from Incyte Corporation. Incyte Corporation (Wilmington, DE, USA) is committed to data sharing that advances science and medicine while protecting patient privacy. Qualified external scientific researchers may request anonymized datasets owned by Incyte for the purpose of conducting legitimate scientific research. Researchers may request anonymized datasets from any interventional study (except Phase 1 studies) for which the product and indication have been approved on or after 1 January 2020 in at least one major market (e.g., US, EU, JPN). Data will be available for request after the primary publication or 2 years after the study has ended. Information on Incyte’s clinical trial data sharing policy and instructions for submitting clinical trial data requests are available at: https://www.incyte.com/Portals/0/Assets/Compliance%20and%20Transparency/clinical-trial-data-sharing.pdf?ver=2020-05-21-132838-960.

## Supplementary Materials

The following are available online at https://www.mdpi.com/1422-0067/22/4/1519/s1, Figure S1: quantitative measurement of anti-CA IgA plasma levels with a commercial ELISA kit validate our results, Figure S2: *Candida albicans* cytokine response is not induced in single cultures of epidermal or CLA^+/−^ T cells, Figure S3: Correlations between CCL18, Chi3L1 and AZU1 plasma levels with disease severity and onset in psoriasis, Table S1: Detailed information on samples from psoriasis and healthy individuals, Table S2: List of antibodies and concentrations used in the ELISA, Table S3: Differentially expressed proteins in psoriasis patients with low versus high anti-CA IgA levels.
