# Supplementary material for: Interplay between Humoral and CLA+ T Cell Response against Candida albicans in Psoriasis
Source: Int J Mol Sci. 2021 Feb 3;22(4):1519. doi: 10.3390/ijms22041519 (PMC7913574; doi:10.3390/ijms22041519)
Supplement: Supplementary file 1 [file ijms-22-01519-s001.pdf]

SUPPORTING INFORMATION

Figure S1. Quantitative measurement of anti-CA IgA plasma levels with a commercial ELISA kit validate our results. Plasma from psoriasis (n=52, ●) and healthy controls (n=15, ○) were tested in *Candida albicans* IgA ELISA kit from IBL-international according to manufacture instructions. Differences in anti-CA IgA titters quantified with the commercial kit were analysed according disease type: plaque (n=31) and guttate (n=21). Dotted line indicates the limit of detection for positive results on *Candida albicans* fresh infection. Mann-Whitney test was used to compare two groups and p values are indicated as *: p<0.05.

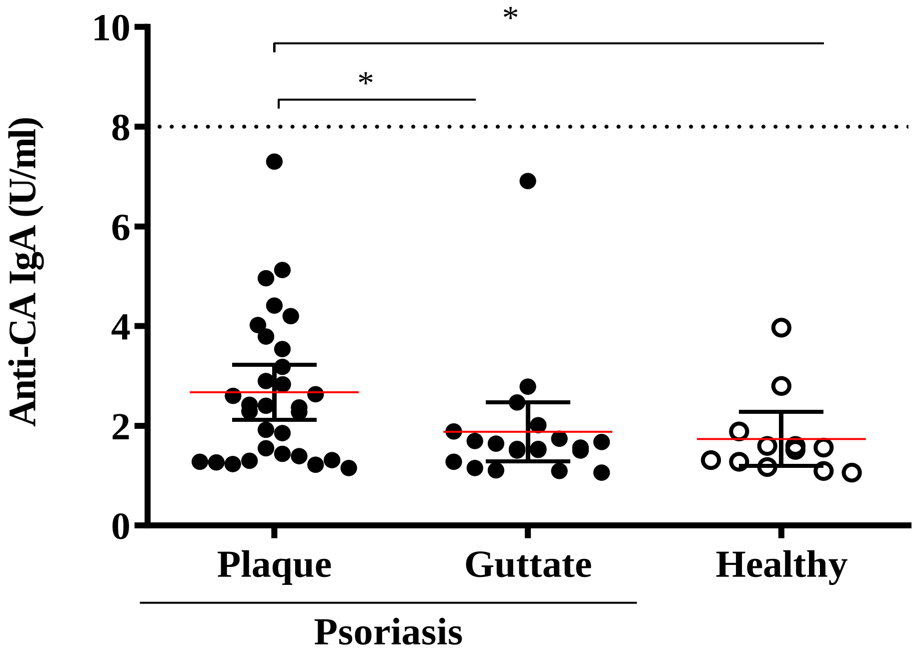

Figure S2. ***Candida albicans*** **cytokine response is not induced in single cultures of epidermal or CLA$^{+/-}$ T cells.** Single cultures of CLA+ T cells, CLA^{-} T cells and epidermal cells were left untreated or stimulated with *C. albicans* extract for 5 days before cytokines were quantified in supernatants (n=4). Levels of IL-17A (a) and IFN-γ (b) were measured. Data are presented as single points for each patient and median (red line).

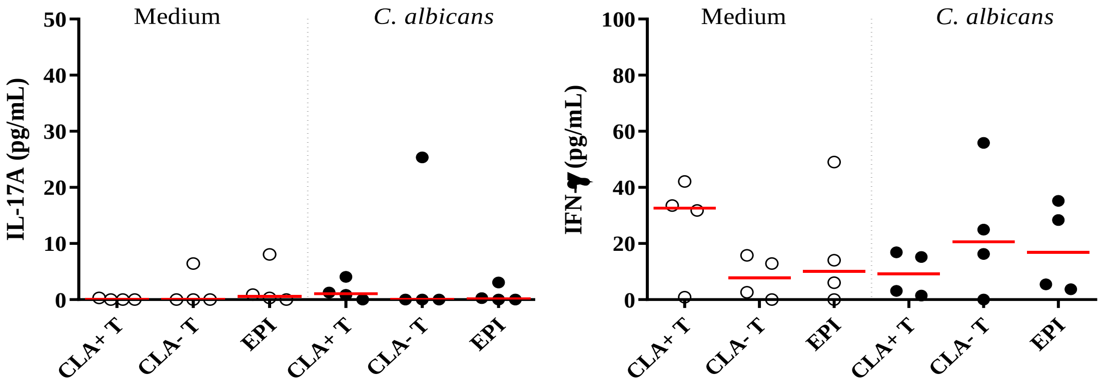

Figure S3. Correlations between CCL18, CHI3L1 and AZU1 plasma levels with disease severity and onset in psoriasis. Levels of CCL18, CHI3L1 and AZU1 were quantified by commercial ELISA kits on plasma from psoriasis patients (n=52). Association with disease severity, in terms of PASI (a-c), and onset (d-f) was assessed by spearman correlation. Spearman coefficient (r) and p values (p) are reported for each condition.

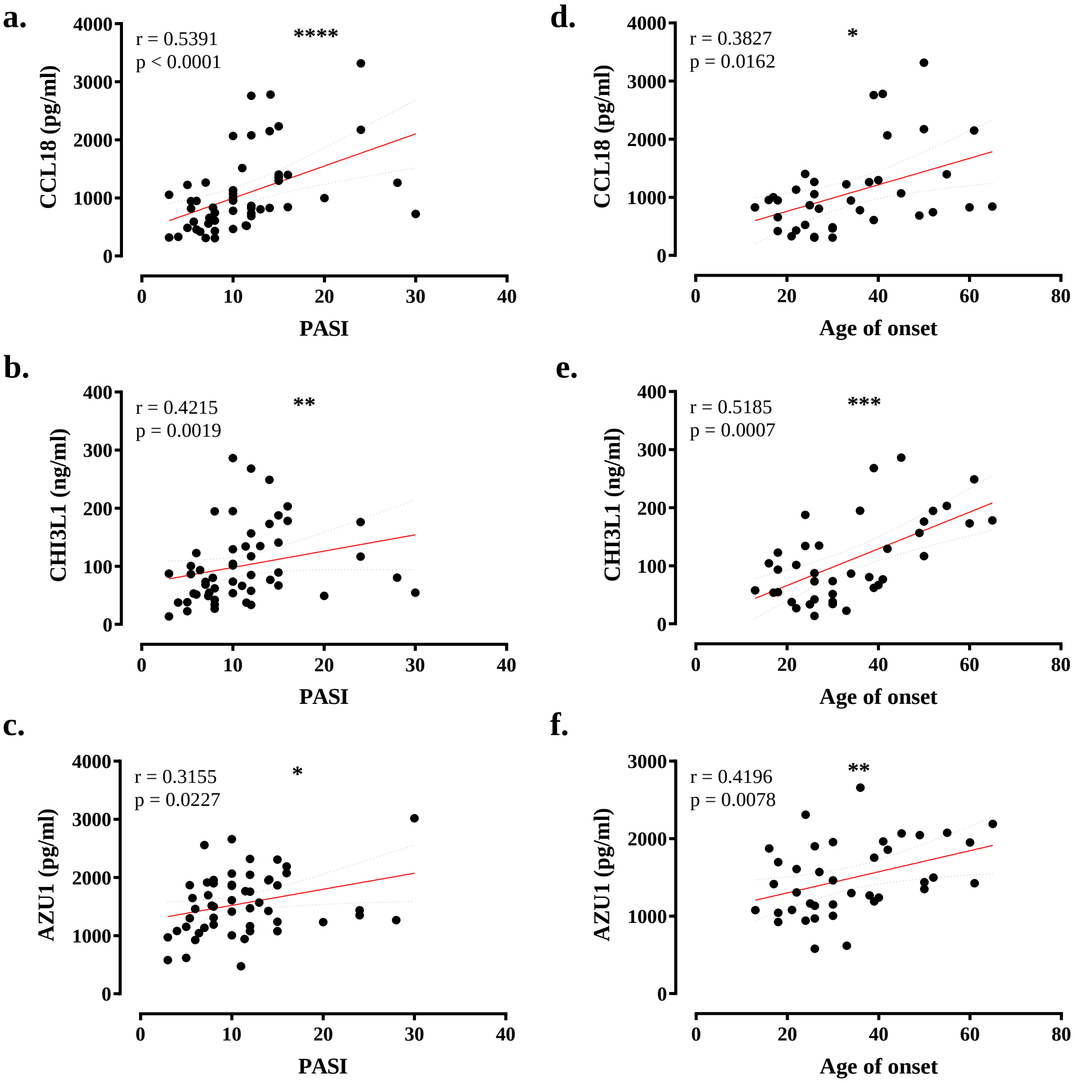

Table S1. Detailed information on samples from psoriasis and healthy individuals. Biopsies from psoriasis patients were performed in skin lesions.

<table>
<tr><th colspan="2" rowspan="2"></th><th colspan="2">Sample type</th><th rowspan="2">Experiments</th><th rowspan="2">Hospital / Institution</th></tr>
<tr><th>Biopsy + blood</th><th>Plasma</th></tr>
<tr><td rowspan="2">Psoriasis (n=166)</td><td>Cohort 1</td><td>52</td><td>52</td><td>• Anti-CA / SE ELISAs
• CLA+/- T cells and EPI cocultures
• ELISAs for validating proteomic results</td><td>- Hospital del Mar, Barcelona (Spain).
- Hospital Arnau de Vilanova de Lleida, Lleida (Spain).
- Iasi (Rumania)</td></tr>
<tr><td>Cohort 2</td><td>-</td><td>114</td><td>• Anti-CA IgA ELISA
• Proteomic profiling</td><td>Incyte Corporation, Clinical Trial NCT00778700</td></tr>
<tr><td colspan="2">Healthy controls (n=17)</td><td>12</td><td>17</td><td>• N=17 Anti-CA/SE ELISAs
• N=12 CLA+/- T cells and EPI cocultures
• N=17 ELISAs for validating proteomic results</td><td>Hospital del Mar, Barcelona (Spain).</td></tr>
</table>

Table S2. List of antibodies and concentrations used in the ELISA.

Antibody name	Company	Reference	Dilution
Anti-human IgA - Alkaline Phosphatase antibody produced in goat	SIGMA-Aldrich	A9669	1:4000
Mouse anti-human IgA1-AP	Southern Biotech	9130-04	1:4000
Mouse anti-human IgA2-AP	Southern Biotech	Sc-17803	1:4000
Anti-Human IgG − Alkaline Phosphatase antibody produced in goat	SIGMA-Aldrich	A9544-.25ML	1:4000
Mouse Anti-Human IgG1 Fc-AP	Southern Biotech	9054-04	1:4000
Mouse Anti-Human IgG2 Secondary antibody, AP	Invitrogen	05-3522	1:500
Mouse Anti-Human IgG3 Secondary antibody, AP	Invitrogen	05-3622	1:1000
Mouse Anti-Human IgG4 Fc-AP	Southern Biotech	9200-04	1:500

Table S3. Differentially expressed proteins in psoriasis patients with low versus high anti-CA IgA levels. Positive fold change values represent higher protein levels in the high IgA-CA group. Asterisks indicate proteins with >30% of values below the limit of detection.

Protein	Gene	Uniprot ID	Fold Change	Raw P-value	FDR P-value
Eosinophil cationic protein	RNASE3	P12724	1.718	0.037	0.978
Azurocidin	AZU1	P20160	1.507	0.035	0.978
Chitinase-3-like protein 1	CHI3L1	P36222	1.401	0.045	0.978
Protein delta homolog 1	DLK1	P80370	1.390	0.01	0.978
C-C motif chemokine 18	CCL18	P55774	1.370	0.006	0.978
Carboxypeptidase B	CPB1	P15086	1.347	0.007	0.978
Ras GTPase-activating protein 1	RASA1*	P20936	1.318	0.032	0.978
Fc receptor-like B	FCRLB	Q6BAA4	1.302	0.009	0.978
NAD kinase 2, mitochondrial	NADK	O95544	1.299	0.041	0.978
Immunoglobulin lambda constant 2	IGLC2	P0CG05	1.274	0.009	0.978
Fc receptor-like protein 5	FCRL5	Q96RD9	1.268	0.011	0.978
Alpha-(1,3)-fucosyltransferase 5	FUT5	Q11128	1.222	0.034	0.978
Trypsin-2	PRSS2	P07478	1.220	0.028	0.978
Procollagen C-endopeptidase enhancer 1	PCOLCE	Q15113	1.217	0.047	0.978
Secreted frizzled-related protein 3	FRZB	Q92765	1.206	0.038	0.978
Angiotensinogen	ANG	P03950	1.191	0.049	0.978
Insulin-like growth factor-binding protein 7	IGFBP7	Q16270	1.167	0.042	0.978
Intercellular adhesion molecule 3	ICAM3	P32942	1.144	0.045	0.978
Beta-1,4-glucuronyltransferase 1	B4GAT1	O43505	-1.117	0.025	0.978
Brevican core protein	BCAN	Q96GW7	-1.171	0.037	0.978
Fas Ligand	FASLG	P48023	-1.175	0.039	0.978
Vascular Endothelial Growth Factor D	VEGFD	O43915	-1.179	0.016	0.978
Receptor for Advanced Glycation Endproducts	AGER	Q15109	-1.192	0.005	0.978
Follistatin	FST	P19883	-1.271	0.011	0.978
Growth differentiation factor 2	GDF2	Q9UK05	-1.377	0.033	0.978
Fibroblast growth factor 2	FGF2*	P09038	-1.430	0.034	0.978
Insulin-like growth factor-binding protein 1	IGFBP1	P08833	-1.601	0.041	0.978
